# Mulberry Fruit Extract Promotes Serum HDL-Cholesterol Levels and Suppresses Hepatic microRNA-33 Expression in Rats Fed High Cholesterol/Cholic Acid Diet

**DOI:** 10.3390/nu12051499

**Published:** 2020-05-21

**Authors:** Soojin Lee, Mak-Soon Lee, Eugene Chang, Yoonjin Lee, Jaerin Lee, Jiyeon Kim, Chong-Tai Kim, In-Hwan Kim, Yangha Kim

**Affiliations:** 1 Department of Nutritional Science and Food Management, Ewha Womans University, Seoul 03760, Korea; alicesujin@naver.com (S.L.); troph@hanmail.net (M.-S.L.); eugenics77@hotmail.com (E.C.); inuyasha_yj@naver.com (Y.L.); dnflwoflssl@naver.com (J.L.); fri0415x2@gmail.com (J.K.); 2 R&D Center, EastHill Corporation, Gwonseon-gu, Suwon-si, Gyeonggi-do 16642, Korea; ctkim@ieasthill.com; 3 Department of Integrated Biomedical and Life Sciences, Korea University, Seoul 02841, Korea; k610in@korea.ac.kr

**Keywords:** bile acid, cholesterol efflux, high-density lipoprotein cholesterol (HDL-C), mulberry fruit, microRNA-33

## Abstract

Serum high-density lipoprotein cholesterol (HDL-C) levels and cholesterol excretion are closely associated with the risk of cardiovascular complications. The specific aim of the present study was to investigate the cholesterol lowering effect of mulberry fruit in rats fed a high cholesterol/cholic acid diet. Four-week supplementation with mulberry fruit extract significantly decreased serum and hepatic cholesterol (TC), serum low-density lipoprotein cholesterol (LDL-C), and fecal bile acid levels without changes in body weight and food intake (*p* < 0.05). Mulberry fruit extract significantly inhibited hepatic sterol-regulatory element binding protein (Srebp) 2 gene expression and upregulated hepatic mRNA levels of liver X receptor alpha (Lxr-α), ATP-binding cassette transporter 5 (Abcg5), and cholesterol 7 alpha-hydroxylase (Cyp7a1), which are involved in hepatic bile acid synthesis and cholesterol metabolism (*p* < 0.05). In addition, hepatic microRNA-33 expression was significantly inhibited by supplementation of mulberry fruit extract (*p* < 0.05). These results suggest the involvement of miR-33, its associated hepatic bile acid synthesis, HDL formation, and cholesterol metabolism in mulberry fruit-mediated beneficial effects on serum and hepatic lipid abnormalities.

## 1. Introduction

Cardiovascular disease (CVD) remains the leading cause of death worldwide [1]. According to the World Health Organization (WHO), the annual number of deaths from CVD will increase up to 22.3 million by 2030, an increase of about 27% compared to 2012 [2]. Dyslipidemia is a major risk factor for CVD, which is characterized by elevated low-density lipoprotein cholesterol (LDL-C) and triglyceride (TG) levels and decreased high-density lipoprotein cholesterol (HDL-C) concentrations [3]. Given a negative correlation between HDL-C levels and the risk of CVD, HDL-C has been demonstrated as a strong predictor of CVD [4,5]. The cardioprotective properties of HDL-C include reverse cholesterol transport from aortic foam cells to the liver, as well as anti-inflammatory, anti-oxidative, and anti-apoptotic effects [6,7]. Thus, it is pivotal to improve HDL formation and function for prevention and/or treatment of CVD.

Cholesterol homeostasis is a complicated process that contains cholesterol biosynthesis, conversion of cholesterol to bile acid, and bile secretion [8]. There are several transcriptional regulators and enzyme activities involved in hepatic cholesterol homeostasis. Under normal physiological status, sterol-regulatory element binding protein (SREBP) 2 is the key transcription factor by regulating numerous genes including 3-hydroxy-3-methylglutaryl-CoA reductase (HMGCR), the rate-limiting enzyme in cholesterol biosynthesis [9]. With excessive dietary cholesterol levels, the nuclear hormone receptor, liver X receptor α (LXRα) regulates ATP-binding cassette protein G5/G8 (ABCG5/ABCG8) in hepatocytes and enterocytes [10,11]. ABCG5/8 contributes to the absorption of excess dietary cholesterol from the intestine and the cholesterol excretion from liver to bile acid to control cholesterol homeostasis [10]. LXRα also regulates cholesterol 7a-hydroxylase (CYP7A1), a rate-limiting enzyme of bile acid biosynthesis that is linked to hepatic cholesterol accumulation and its serious consequence, CVD [12]. Bile acids bind to farnesoid X receptor (FXR), and induces FXR activation, all of which stimulate the FXR target gene, small heterodimer partner (SHP) expression in the liver. Bile acid-induced FXR promotes bile secretion by increasing expression of ATP-binding cassette subfamily B, member 11 (ABCB11), known as the bile salt export pump, and ATPase phospholipid transporting 8B1 (ATP8B1), and by reducing bile acid synthesis through the inhibition of CYP7A1 expression [13]. For cellular cholesterol efflux, excess cholesterol from peripheral cells and tissues is transferred to HDL particles, which transport it back to the liver for concomitant disposal into the bile acids. Fecal loss of bile acid is compensated by hepatic bile acid synthesis, leading to hepatic cholesterol depletion [14]. Increasing HDL-C levels has been regarded as a therapeutic approach to prevent CVD by inhibiting LDL oxidation and protecting endothelial cells from oxidized LDL-induced cytotoxic effects [5]. The main regulators of HDL formation, ATP-binding cassette transporter A1 (ABCA1), apolipoprotein A-I (apoA-I), and lecithin and cholesterol acyltransferase (LCAT) are also inversely related to CVD. ABCA1 has been identified as the rate-limiting transporter for HDL formation and maturation as well as cholesterol efflux [15]. ApoA-I, a major structural protein of HDL particles, regulates HDL-C biosynthesis and shows a positive correlation with blood HDL-C levels [16]. LCAT is a critical enzyme for HDL particle maturation and cholesterol efflux [17]. Improving cholesterol metabolism and bile acid synthesis while raising HDL cholesterol appears to be an attractive therapeutic strategy to reduce CVD risk.

Mulberry (*Morus alba* L) belongs to the genus Morus in the Moraceae family. The flowers of *Morus alba* L are called Sang Shen and Oddi [18]. Numerous studies demonstrate various biological activities of mulberry, including anti-oxidant [19], anti-diabetic [20], and anti-inflammatory properties [21]. In addition, many biologically active components have been reported, including anthocyanins and flavonols [18,22]. High hydrostatic pressure (HHP), a recent non-thermal food processing technique, has been developed to extract bioactive compounds without thermally destroying the activity and structure of bioactive components [23]. A study illustrates that the HHP procedure maintains higher total phenolic, flavonoid, and resveratrol levels and anti-oxidant activity of mulberry juice than the heat treatment process [24]. Therefore, the purpose of the present study was to investigate if HHP-treated mulberry fruits improve serum HDL-C concentrations as well as other serum lipid markers in rats fed a high cholesterol/cholic acid diet. This was done by measuring serum metabolic profiles, hepatic expression of genes involved in bile acid synthesis, cholesterol synthesis and efflux, HDL formation, and hepatic miR-33 expression.

## 2. Materials and Methods

### 2.1. Preparation of High Hydrostatic Pressure-Treated Mulberry Fruit Extract

HHP extract of mulberry fruits was prepared by the Korea Food Research Institute (KFRI; Wanju, Korea). Frozen mulberry fruits (*Morus alba* L) from the Sangju silkworm farming association (Sangju, Geongsangbuk-do, Korea) were chopped into small particles, homogenized in a Waring blender for 5 min, and mixed with 40,000 units each of Pectinex ultra color. Then, Pectinex BE XXL enzymes (Daejong Trade Co. Seoul, Korea) were poured into plastic bags and a high pressure machine (TFS-50L, Innoway Co., Bucheon, Korea) under 100 Mpa at 50 °C was utilized. After 4 h extraction, the extracts were boiled for 10 min for inactivation. After cooling, the extracts were centrifuged at 11,000× *g* for 5 min, filtered using Whatman No. 5 filter paper, and freeze-dried until use.

### 2.2. Animals and Experimental Design

Six-week old male Sprague Dawley rats weighing 180–200 g were purchased from Doo Yeol Biotech (Seoul, Korea). Each rat was housed in an individual rack under a controlled environment of 12-h light/dark cycle with constant temperature (22 ± 2 °C) and humidity (55% ± 5%). All rats were acclimatized for 1 week with free access to water and normal chow diet (Harlan 2018S rodent diet, Harlan, USA) and were randomly divided into three groups (*n* = 6/group) as follows: normal diet (NOR, Harlan 2018S rodent diet, Harlan, USA), high cholesterol/cholic acid diet containing 1% cholesterol and 0.5% cholic acid (HC), and HC containing 0.4% HHP-treated mulberry (HM). The diet compositions of NOR, HC, and HM were described in Supplementary Table S1. Body weights and food intake were measured twice a week during the 4-week experimental period. During the last three consecutive days before the end of the experiment, feces were collected and stored at −40 °C until further use. After 12 h of overnight fasting, rats were anesthetized with an intraperitoneal injection of a mixture of Zoletil 50 (Virbac Laboratories, Carros, France) and Rompun (Bayer Korea, Seoul Korea). Blood samples were collected by cardiac puncture, separated by centrifugation at 1516× *g* for 20 min, and stored at −40 °C until use. Excised liver and epididymal white adipose tissue (eWAT) samples, except for the histological analysis sample, were immediately frozen in liquid nitrogen and stored at −70 °C for further analysis. All experimental procedures were approved by the Institutional Animal Care and Use Committee (IACUC) of Ewha Womans University (IACUC No. 18-011).

### 2.3. Determination of Serum Metabolic Parameters

Serum activities of alanine transaminase (ALT) and aspartate transaminase (AST) and serum concentrations of TG, total cholesterol (TC), and HDL-C were determined by enzymatic colorimetric methods using commercially available kits (Asan Pharmaceutical Co., Seoul, Korea). Serum contents of LDL-C were calculated using the Friedewald formula: (LDL-C = TC − HDL-C − (TG/5)) [25].

### 2.4. Hepatic and Fecal Lipid Analysis

Hepatic and fecal lipid extraction were conducted using the method of Bligh and Dyer as previously described [26]. A total of 0.5 g of wet liver tissues were homogenized in 1.5 mL of 0.9% saline. Then, 7.5 mL of chloroform and methanol mixture (1:2, v/v) were added to the homogenates. After 10 min of vortexing and 1 h of stability, 2.5 mL of chloroform were added and centrifuged at 2214× *g* for 20 min. Using a Pasteur pipette, the clear lower phase was moved to a fresh tube, filtered through a Whatman No.6 filter paper, dried, and weighed. A mixture of n-hexane/isopropanol (3/2, v/v) was used to dissolve lipid extract. For fecal lipid extraction, feces were dried at 65 °C for one day, ground, and weighed. Fecal lipids were extracted in the same way that the liver lipids were extracted as described above. As previously described, enzymatic colorimetric methods were used to determine hepatic and fecal TG and TC levels.

### 2.5. Fecal Bile Acid Analysis

A total bile acid-test kit (Wako, Osaka, Japan) was utilized to measure fecal total bile acids (TBA) according to the manufacturer’s instruction. First, fecal lipid extraction was executed as described above. Using the enzymatic calorimetric method, the reacted substrates create end color products that are directly proportional to fecal TBA contents. Absorbance was measured at a wavelength of 560 nm by a Varioskan plate reader (Thermo Scientific, Waltham, MA, USA) and expressed as fold change with respect to the HC group.

### 2.6. Histological Analysis

Dissected liver tissues were fixed in 10% formalin buffer overnight at room temperature. The fixed liver tissues were then embedded in paraffin blocks, sliced into 6 μm thick sections by a microtome (Leica-microsystems, Wetzlar, Germany), and stained with hematoxylin-eosin (H&E). Digital images of stained liver tissue sections were captured with an Olympus IX 51 inverted microscope (Olympus, Tokyo, Japan) at 200× magnification.

### 2.7. Real-Time Quantitative Polymerase Chain Reaction (RT-qPCR)

A Ribo Ex total RNA solution (Geneall Biotechnology Co., Daejeon, Korea) was used to isolate total RNA from liver tissues according to the manufacture’s instruction. The concentration and purity of isolated RNA were determined by a spectrophotometer (Ultrospec 2100 pro, Amersham Bioscience, Sweden). The integrity of RNA was confirmed by staining two ribosomal RNA bands (18S and 28S rRNA) with ethidium bromide (EtBr) and electrophoresed on 1.5% agarose gel. Isolated RNA was reverse transcribed to cDNA using a moloney murine leukemia virus reverse transcriptase (MMLV RTase, Bioneer Co., Daejeon, Korea). Real-time quantitative polymerase chain reaction (RT-qPCR) was performed using a fluorometric thermal cycler (Rotor GeneTM 3000; Corbett Research, Mortlake, NSW., Australia) and AccuPower 2X Greenstar qPCR Master mix (−ROX Dye) (Bioneer Co.). To optimize conditions for each primer pair, the PCR efficiency, including slope, R^2^, and melt curve, was analyzed using a serial dilution of pooled samples. In addition, electrophoresis with 1.5% agarose gel and PCR reaction products was conducted to check out primer dimer production. Primers used in the current study are listed in Supplementary Table S2. Gene expression was normalized to the housekeeping gene, β-actin, calculated using the ΔΔCt method [27] and expressed as fold difference compared to the HC group.

A miRNA cDNA Synthesis Kit with Poly (A) Polymerase Tailing (ABM Inc., Richmond, BC, Canada), EvaGreen miRNA qPCR Master Mix (ABM Inc.), and specific primers for miR-33 and U6 (ABM Inc.) were utilized to measure hepatic microRNA expression. After RT-qPCR amplification using the Rotor Gene 3000 (Corbett Research), hepatic miR-33 expression was normalized to U6 snRNA and expressed as fold change compared to the HC group.

### 2.8. Statistical Analysis

Data are expressed as mean ± standard error of the mean (SEM). Statistical differences between two or three groups were determined using the one-tailed Student’s t-test or one-way analysis of variance (ANOVA) followed by Tukey’s multiple comparison tests using SPSS software (version 23; IBM Corporation, Armonk, NY, USA). Significant differences were determined at *p* < 0.05.

## 3. Results

### 3.1. Effects of Mulberry Fruit Extract on Body Weight, Food Intake, and Adipose Tissue Mass

At 4 weeks of supplementation, there were no statistical changes in final body weight, body weight gain, food intake, energy intake, and food efficiency among all experimental groups. Moreover, no statistical difference in eWAT mass was measured (Table 1).

### 3.2. Effects of Mulberry Fruit Extract on Liver Weight and Serum AST and ALT Activities

Four weeks of consumption of the HC diet significantly increased liver weight by 36.5% compared to the NOR group, resulting in HC-induced hepatomegaly. However, no further change was found with supplementation of the HC diet with mulberry fruit extract (Table 1). Serum AST and ALT activities were measured to investigate the possibility that a HC diet supplemented with HHP extract of mulberry fruits contributes to liver toxicity. Compared to the HC diet, HHP-treated mulberry fruit extract did not change serum AST and ALT activities, showing that 0.4% mulberry fruit in HC diet was tolerated well by rats (Table 1).

### 3.3. Effects of Mulberry Fruit Extract on Serum Lipid Parameters

**Figure 1:**
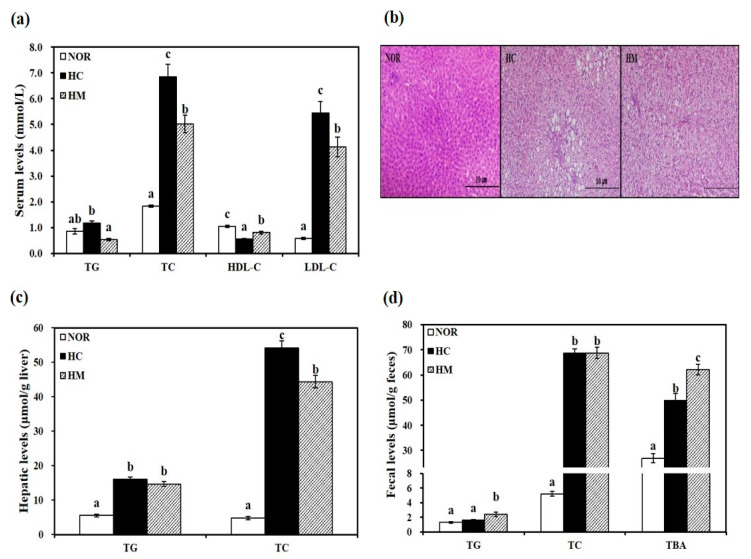
Influence of high hydrostatic pressure mulberry fruit extract on serum (**a**), hepatic (**c**), and fecal lipid profiles (**d**). (**b**) Representative hematoxylin and eosin (H&E) stained liver tissue (scale bars = 100 μm; magnification of 200×). Data are expressed as mean ± SEM (*n* = 6). Bars with different letters (a, b, c) are statistically different at *p* < 0.05. HDL-C, HDL-cholesterol; LDL-C, LDL-cholesterol; TBA, total bile acid; TC, total cholesterol; TG, triglyceride; NOR, normal diet; HC, high-cholesterol/cholic acid diet containing 1% cholesterol and 0.5% cholic acid; and HM, HC + 0.4% high hydrostatic pressure mulberry fruit extract.

Serum levels of TG, TC, LDL-C, and HDL-C were measured to investigate the effect of a HC diet supplemented with mulberry fruit extract on HC-induced dyslipidemia. Rats fed a HC diet showed higher serum TG, TC, and LDL-C contents than animals fed a NOR diet. Supplementation with mulberry fruit extract significantly decreased serum TG, TC, and LDL-C concentrations by 54.3%, 26.8%, and 24.1%, respectively, compared to the HC group (*p* < 0.05, Figure 1a). Interestingly, rats in the HM group showed higher serum HDL-C levels than the HC group (*p* < 0.05, Figure 1a), indicating the beneficial effect of mulberry extract on HC-decreased HDL-C levels, a powerful predictor of CVD.

### 3.4. Effects of Mulberry Fruit Extract on Hepatic Lipid Profiles

At the end of a 4-week experimental period, the HC diet induced significant increases of hepatic TG and TC levels with larger lipid droplets in hepatocytes, as observed by H&E staining (*p* < 0.05, Figure 1b,c). Decreased hepatic TG levels in the HM group were observed without statistical difference. HC-increased hepatic TC levels were significantly inhibited by 18.2% with mulberry fruit extract supplementation (*p* < 0.05, Figure 1c).

### 3.5. Effects of Mulberry Fruit Extract on Fecal Lipid Profiles and Bile Acid Excretion

Next, fecal levels of TG, TC, and TBA were measured. After 4 weeks of diet intervention, fecal TG levels in the HC group were not statistically different compared to the NOR group. However, the HM group had 50.6% higher fecal TG levels than the HC group (*p* < 0.05, Figure 1d). There was no statistical difference in fecal TC concentrations between HC and HM groups (Figure 1d). As shown in Figure 1d, fecal bile acid excretion as measured by TBA levels in the HC group was statistically higher than in the NOR group. Four-week supplementation with mulberry fruit extract significantly increased fecal TBA levels by 24.5% compared to the HC diet (*p* < 0.05, Figure 1d).

### 3.6. Effect of Mulberry Fruit Extract on Hepatic Gene Expression related to Cholesterol Metabolism and Bile Acid Synthesis

**Figure 2:**
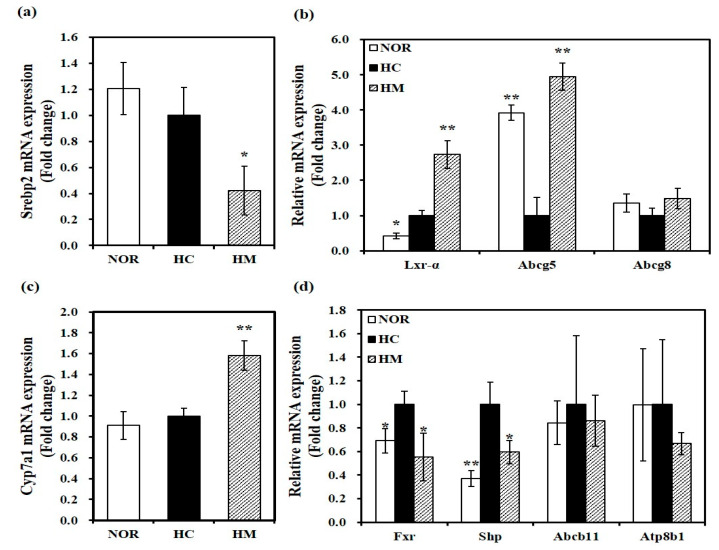
Effect of high hydrostatic pressure mulberry fruit extract on mRNA levels related to cholesterol homeostasis in the liver. Hepatic mRNA levels of Srebp2 (**a**); liver X receptor-α (Lxr-α), ATP-binding cassette sub-family G member 5 (Abcg5), and Abcg8 (**b**); cholesterol 7 alpha-hydroxylase (Cyp7a1) (**c**); and farnesoid X receptor (Fxr), small heterodimer partner (Shp), and ATP-binding cassette subfamily B, member 11 (Abcb11) (**d**) were measured by RT-qPCR and normalized to β-actin. Data were expressed as fold change compared to the HC and expressed as mean ± SEM (*n* = 6). * *p* < 0.05; ** *p* < 0.01 compared to the HC diet. NOR, normal diet; HC, high cholesterol/cholic acid diet containing 1% cholesterol and 0.5% cholic acid; and HM, HC + 0.4% high hydrostatic pressure mulberry fruit extract.

To investigate the effect of HHP-treated mulberry fruit extract on mRNA levels involved in cholesterol homeostasis, hepatic mRNA levels were analyzed by qRT-PCR. As shown in Figure 2a, 54% significant reduction of hepatic Srebp2 mRNA expression related to de novo cholesterol biosynthesis was measured in rats fed a HM diet (*p* < 0.05). Compared to the HC diet, a HC diet supplemented with HHP-treated mulberry fruit extract significantly upregulated hepatic gene expression of Lxr-α and Abcg5, key transcriptional regulators of cholesterol efflux (*p* < 0.05). Although not statistically different, a 1.48-fold increase of hepatic Abcg8 mRNA expression was observed in the HM group compared to the HC group (Figure 2b). Thus, we suggest that the hypolipidemic effect of mulberry fruits might be involved in changes in mRNA transcription related to cholesterol synthesis and efflux. In the liver, mRNA expression of Cyp7a1, a rate-limiting enzyme in bile acid synthesis, was significantly enhanced by about 1.6-fold in rats fed a HM diet compared to the HC diet (*p* < 0.05, Figure 2c). In addition, hepatic mRNA levels of Fxr and Shp were significantly increased by the high cholesterol/cholic acid diet (HC), which was downregulated significantly by supplementation of mulberry fruit extract in the HC diet (*p* < 0.05, Figure 2d). However, Fxr-target gene, Abcb11, and Atp8b1 gene expression were not changed by either the HC diet or supplementation of mulberry fruit extract (Figure 2d).

### 3.7. Effect of Mulberry Fruit Extract on Hepatic Gene Expression related to HDL Formation

**Figure 3:**
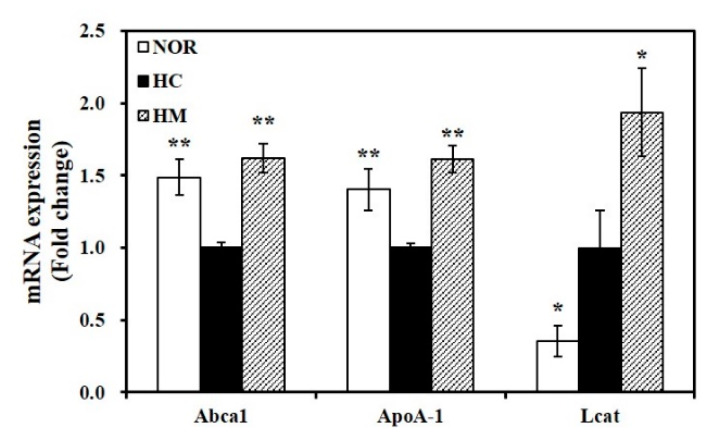
Effect of high hydrostatic pressure mulberry fruit extract on hepatic mRNA levels involved in HDL formation. ATP-binding cassette, subfamily A member 1 (Abca1), apolipoprotein A-1 (ApoA-1), and lecithin cholesterol acyltransferase (Lcat) mRNA levels were measured by RT-qPCR and normalized to β-actin. Data were expressed as fold change compared to the HC and are expressed as mean ± SEM (*n* = 6). * *p* < 0.05; ** *p* < 0.01 compared to the HC diet. NOR, normal diet; HC, high cholesterol/cholic acid diet containing 1% cholesterol and 0.5% cholic acid; and HM, HC + 0.4% high hydrostatic pressure mulberry fruit extract.

Hepatic mRNA levels of Abca1, ApoA-1, and Lcat were measured to examine the influence of mulberry fruit extract on serum HDL-C levels. HC-reduced Abca1 and ApoA-1 hepatic mRNA levels were significantly upregulated by mulberry fruit extract (*p* < 0.05, Figure 3). In addition, 4-week supplementation with HHP-treated mulberry fruit extract in the HC diet significantly increased Lcat gene expression by 1.9-fold (*p* < 0.05, Figure 3), suggesting that supplementing the HC diet with mulberry fruit extract enhances HDL formation.

### 3.8. Effect of Mulberry Fruit Extract on Hepatic miR-33 Expresion

**Figure 4:**
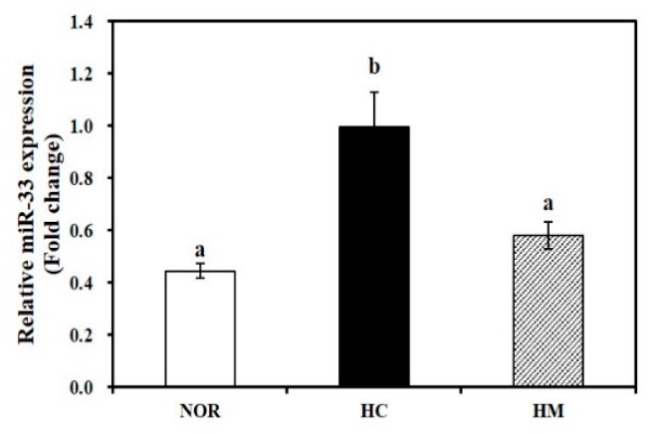
Effect of high hydrostatic pressure mulberry fruit extract on hepatic miR-33 expression. Hepatic miR-33 levels were measured by RT-qPCR, normalized to U6 snRNA, expressed as the fold change compared to the HC group, and expressed as mean ± SEM (*n* = 6). Mean values with different superscript (a, b) are significantly different at *p* < 0.05. NOR, normal diet; HC, high cholesterol/cholic acid diet containing 1% cholesterol and 0.5% cholic acid; and HM, HC + 0.4% high hydrostatic pressure mulberry fruit extract.

To demonstrate the molecular mechanism by which HHP-treated mulberry fruit extract controls cholesterol homeostasis and increases HDL formation, hepatic miR-33 expression was measured. As shown in Figure 4, the HC diet significantly increased hepatic miR-33 expression by 55.2% compared to the NOR diet (*p* < 0.05). Four-week supplementation of the HC diet with mulberry fruit extract significantly downregulated HC-induced miR-33 expression by 41.5% (*p* < 0.05).

## 4. Discussion

Accumulating epidemiologic and prospective evidence has demonstrated that lower circulating HDL-C is negatively associated with an increased risk of CVD [4,5,28]. In addition to HDL-cholesterol efflux capacity, HDL also exerts anti-oxidative, anti-inflammatory, and anti-apoptotic properties and restores endothelial function, all of which contribute to decreased risk of CVD [6,7]. Therefore, understanding the features of HDL formation, dysfunction, and capacity might allow development of therapeutic approaches to CVD. Growing evidence has shown that mulberry fruits have anti-oxidative and anti-inflammatory actions [19,21,29,30]. HHP was recently introduced as a non-thermal food processing technique that prevents degradation of activity and structure of bioactive components [23]. This information led us to investigate the beneficial effect of HHP extract of mulberry fruit extract on HC-induced changes in HDL formation and function. We elucidated the favorable effect of mulberry fruit on serum and hepatic lipid profiles by measuring mRNA levels involved in cholesterol efflux and bile acid synthesis as well as hepatic miR-33 expression.

In the current study, male Sprague-Dawley rats were fed NOR, HC, or HM diets for 4 weeks. The dose of mulberry fruit extract given in the HM diet was determined as described in previous studies [31,32]. The 0.4% HHP-treated mulberry fruit extract in the HC diet was tolerated well by rats, as indicated by no statistical difference in serum ALT and AST activities and liver weight compared to rats fed a HC diet. Similar to previous studies using aqueous or ethanol extraction or freeze-dried powder of mulberry fruits [31,32,33], HHP extraction of mulberry fruits significantly decreased serum TG, TC, and LDL-C levels. Based on our previous study, HHP-treated mulberry fruits contain anthocyanins, including cyanidin 3-O-glucoside and cyanidin 3-O-rutinoside and flavonols such as quercetin and quercetin 3-O-rutinoside [21]. Isolated and purified anthocyanin from mulberry fruits, anthocyanin-rich pomegranate juice, and quercetin supplementation improve serum lipid profiles [34,35,36]. Therefore, we speculate that the favorable effect of HM on serum lipid parameters might be involved in its major bioactive compounds such as anthocyanins and flavonols. As previously reported using virgin olive oil or cocoa [37], other biomaterials or polyphenol-rich nutrients might be associated with the hypolipidemic properties of HHP-mulberry fruits.

The negative effect of nonalcoholic fatty liver disease (NAFLD) on CVD risk is associated with the abnormalities in cardiac metabolism and function [38]. In the present study, a 4-week HC diet significantly increased serum and hepatic TG and TC levels and larger lipid droplets in hepatocytes, as observed by H&E staining, when compared to the NOR diet. Supplementation of mulberry fruit extract in the HC diet significantly suppressed hepatic TC levels with reduction of serum TG and TC levels without changing hepatic mRNA levels involved in inflammation (data not shown). Thus, HM-decreased serum and hepatic lipids might implicate the favorable effects of mulberry fruits on the initial stage of NAFLD. A following study to investigate how chronic administration of mulberry fruit extract influences fatty liver/liver injury might need to be conducted.

Hepatic de novo cholesterol biosynthesis, uptake, and secretion and bile acid synthesis and secretion are involved in hepatic cholesterol homeostasis [8]. Biosynthesis of bile acids, the final products of cholesterol catabolism, and their excretion to feces contribute to reduction of excess hepatic cholesterol [14,39]. In the present study, rats fed a HC diet with HHP-treated mulberry fruit extract showed lower hepatic TC levels and higher fecal bile acid contents when compared to the HC diet. In addition, mulberry fruit extract significantly inhibited hepatic Srebp2 gene expression and upregulated hepatic Lxr-α, Abcg5, and Cyp7a1 mRNA levels. SREBP2 overexpression increases transcripts of enzymes related to cholesterol biosynthesis and HMGCR [9,40]. A major regulator of dietary cholesterol, LXRα reduces cholesterol absorption and stimulates biliary cholesterol secretion by regulating hepatic and intestinal Abcg5/8 gene transcripts [10]. Genetic ablation of Abcg5/8 upregulates cholesterol absorption and inhibits biliary cholesterol concentrations [41]. In addition, LXRα regulates CYP7A1, a key enzyme for bile acid formation and cholesterol excretion [12]. Decreased bile acid excretion and hypercholesteremia were observed in a proband’s family with CYP7A1 deficiency [42]. Increased CYP7A1 activity promotes hepatic bile acid biogenesis and biliary cholesterol secretion [43]. In the feedback of bile acid regulation, inhibition of CYP7A1 and its-associated decrement in bile acid synthesis result from binding of bile acid to Fxr, its activation, and SHP induction. In the liver, FXR leads to bile acid excretion by ABCB11 and ATP8B1. Hepatic ABCB11 overexpression increases Fxr/Shp expression and decreases hepatic Cyp7a1 expression, which in turn inhibits bile acid synthesis [44]. ATP8B1 deletion enhances biliary output of cholesterol by inhibiting Fxr mRNA and protein expression and decreasing Fxr target genes, Abcb11, and Shp expression [45,46]. In our present study, high cholesterol/cholic acid diet significantly upregulated hepatic mRNA levels of Fxr and Shp, which were suppressed by HHP-treated mulberry fruit extract. However, HC diet or supplementation of mulberry fruit extract did not change Fxr target, Abcb11, and Atpb81 transcripts related to bile acid export. This suggests that the cholesterol lowering effect of HHP-treated mulberry fruit extract might, at least in part, interact with the nuclear hormone receptor, Lxr-α and its associated Abcg5 and Cyp7a1 gene transcripts involved in cholesterol efflux and bile acid synthesis. A following study is necessary to delineate the molecular mechanisms by which mulberry fruit extract influences bile acid transport and metabolism in the liver and intestine.

HDL-cholesterol efflux capacity from macrophages via ABCA1 and ABCG1 pathways related to reverse cholesterol transport is a major advantage of HDL function associated with lowering CVD [5,47,48]. The nuclear hormone receptor Lxr-α promotes Abca1 transcription [49]. ABCA1 facilitates cholesterol efflux to lipid-free or lipid-poor apoA-I, a major apolipoprotein of HDL, leading to nascent HDL formation that contributes to HDL levels [16]. Overexpression of ABCA1 in mice shows the atheroprotective action of ABCA1 [50,51]. Overexpression of apoA-I also enhances HDL levels and prevents plaque progression [52]. By promoting cholesterol partitioning from the surface to the core, changing small discoidal HDLs to larger spherical HDL particles, and preventing cholesterol export from HDL, LCAT is responsible for HDL formation and maturation and reverse cholesterol transport [17]. Genetic mutations in ABCA1, apoA-1, and LCAT are associated with familial hypoalphalipoproteinemia. Subjects with theses mutations and significant reduction of HDL-C have increased risk of coronary artery disease [53]. In the current study, rats fed a HC diet supplemented with mulberry fruit extract had significantly higher serum HDL-C concentrations and hepatic mRNA levels of Abca1, ApoA-1, and Lcat when compared to animals with the HC diet. These results demonstrate that HHP-treated mulberry fruit extract might enhance serum HDL-C levels by modulating hepatic gene expression related to HDL formation.

MicroRNAs (miRNAs) are single-stranded small RNA molecules (18–25 nucleotides long) that post-transcriptionally regulate physiologic processes by binding to complementary target sites in the 3′ untranslated regions (3′UTRs) of mRNAs [54]. miR-33, an intronic miRNA located within the gene encoding SREBF-2, is a transcriptional regulator of cholesterol synthesis that controls gene transcripts involved in cholesterol homeostasis [55,56,57]. In the current study, supplementation of mulberry fruit extract in HC diet significantly decreased hepatic Srebp2 mRNA expression. Hepatic overexpression of miR-33 [55] and lentiviral delivery of miR-33 [57] decrease hepatic Abca1 expression involved in cholesterol efflux to apoA-1 and blood HDL-C concentration [55]. In contrast, inhibition of miR-33 promotes Abca1 expression and cholesterol efflux and enhances plasma HDL levels [56], indicating that miR-33 might regulate HDL formation and cholesterol efflux. In the present study, we found for the first time that HHP-treated mulberry fruit extract significantly suppressed hepatic Srebp2 and miR-33 expression. The results show that mulberry fruit extract increases HDL formation by regulating hepatic miR-33 levels. Still, further investigation with female animals is needed to demonstrate how mulberry fruit extract influences serum and hepatic lipid metabolism in women.

## 5. Conclusions

In the present study, the favorable effects of HHP-treated mulberry fruit extract on serum HDL-C levels and serum and hepatic lipid abnormalities were investigated in rats fed a high cholesterol diet. Four-week supplementation with mulberry fruit extract suppressed HC diet-induced serum and hepatic lipid parameters and enhanced fecal lipid excretion. Significant induction of serum HDL-C levels was found in rats fed a HC diet containing mulberry fruit. Moreover, HHP-treated mulberry fruit extract significantly increased hepatic mRNA levels related to cholesterol efflux, bile synthesis, and HDL formation. Given the close association between miR-33 and post-translational regulation, we speculate that the beneficial effects of mulberry fruit might be related to miR-33 expression and associated cholesterol metabolism, bile acid synthesis, and HDL formation.

## Figures and Tables

**Table 1:** Effect of mulberry fruit extract on physiological variables.

Variables	NOR	HC	HM
Initial body weight (g)	243.09 ± 3.18	242.18 ± 1.88	240.57 ± 1.26
Final body weight (g)	402.00 ± 6.70	404.97 ± 4.87	406.95 ± 4.59
Body weight gain (g/4 week)	158.90 ± 6.18	162.79 ± 4.83	166.39 ± 5.17
Food intake (g/day)	24.03 ± 0.49	24.06 ± 0.56	25.14 ± 0.19
Energy intake (kcal/day)	90.67 ± 1.83	89.37 ± 2.07	92.97 ± 0.71
Food efficiency ^1)^	0.24 ± 0.01	0.24 ± 0.00	0.24 ± 0.01
Epididymal fat weight (g/100g body weight)	1.52 ± 0.10	1.39 ± 0.06	1.37 ± 0.03
Liver weight (g/100g body weight)	3.28 ± 0.06 ^a^	5.17 ± 0.09 ^b^	5.46 ± 0.18 ^b^
Serum ALT (IU/L)	6.02 ± 0.25	8.75 ± 0.95	7.13 ± 0.91
Serum AST (IU/L)	35.88 ± 1.50 ^a^	41.54 ± 2.55 ^ab^	45.47 ± 3.00 ^b^

^1)^ Food efficiency = body weight gain (g/day)/food intake (g/day); data are expressed as mean ± SEM (*n* = 6). Values with different letters are significantly different at *p* < 0.05. NOR, normal diet; HC, high cholesterol/cholic acid diet containing 1% cholesterol and 0.5% cholic acid; and HM, HC + 0.4% high hydrostatic pressure mulberry fruit extract.

## Supplementary Materials

The following are available online at https://www.mdpi.com/2072-6643/12/5/1499/s1, Table S1: The compositions of experimental diets, Table S2: Primers used for quantitative real-time polymerase chain reaction (qRT-PCR).

## References

[1] Barquera S., Pedroza-Tobias A., Medina C., Hernandez-Barrera L., Bibbins-Domingo K., Lozano R., Moran A.E. (2015). Global Overview of the Epidemiology of Atherosclerotic Cardiovascular Disease. Arch. Med. Res..

[2] World Health Organization (2016). Hearts: Technical Package for Cardiovascular Disease Management in Primary Health Care.

[3] Stone N.J., Robinson J.G., Lichtenstein A.H., Bairey Merz C.N., Blum C.B., Eckel R.H., Goldberg A.C., Gordon D., Levy D., Lloyd-Jones D.M. (2014). 2013 ACC/AHA guideline on the treatment of blood cholesterol to reduce atherosclerotic cardiovascular risk in adults: A report of the American College of Cardiology/American Heart Association Task Force on Practice Guidelines. J. Am. Coll. Cardiol..

[4] Gordon D.J., Rifkind B.M. (1989). High-density lipoprotein—The clinical implications of recent studies. N. Engl. J. Med..

[5] Barter P., Gotto A.M., LaRosa J.C., Maroni J., Szarek M., Grundy S.M., Kastelein J.J., Bittner V., Fruchart J.C. (2007). HDL cholesterol, very low levels of LDL cholesterol, and cardiovascular events. N. Engl. J. Med..

[6] Rye K.A., Barter P.J. (2014). Regulation of high-density lipoprotein metabolism. Circ. Res..

[7] Rosenson R.S., Brewer H.B., Ansell B.J., Barter P., Chapman M.J., Heinecke J.W., Kontush A., Tall A.R., Webb N.R. (2016). Dysfunctional HDL and atherosclerotic cardiovascular disease. Nat. Rev. Cardiol..

[8] Groen A.K., Bloks V.W., Verkade H., Kuipers F. (2014). Cross-talk between liver and intestine in control of cholesterol and energy homeostasis. Mol. Asp. Med..

[9] Madison B.B. (2016). Srebp2: A master regulator of sterol and fatty acid synthesis. J. Biol. Chem..

[10] Repa J.J., Berge K.E., Pomajzl C., Richardson J.A., Hobbs H., Mangelsdorf D.J. (2002). Regulation of ATP-binding cassette sterol transporters ABCG5 and ABCG8 by the liver X receptors alpha and beta. J. Biol. Chem..

[11] Zelcer N., Hong C., Boyadjian R., Tontonoz P. (2009). LXR regulates cholesterol uptake through Idol-dependent ubiquitination of the LDL receptor. Science.

[12] Peet D.J., Turley S.D., Ma W., Janowski B.A., Lobaccaro J.M., Hammer R.E., Mangelsdorf D.J. (1998). Cholesterol and bile acid metabolism are impaired in mice lacking the nuclear oxysterol receptor LXR alpha. Cell.

[13] Sarenac T.M., Mikov M. (2018). Bile Acid Synthesis: From Nature to the Chemical Modification and Synthesis and Their Applications as Drugs and Nutrients. Front. Pharmacol..

[14] Rohrl C., Stangl H. (2018). Cholesterol metabolism-physiological regulation and pathophysiological deregulation by the endoplasmic reticulum. Wien. Med. Wochenschr..

[15] Oram J.F., Heinecke J.W. (2005). ATP-binding cassette transporter A1: A cell cholesterol exporter that protects against cardiovascular disease. Physiol. Rev..

[16] Wang S., Gulshan K., Brubaker G., Hazen S.L., Smith J.D. (2013). ABCA1 mediates unfolding of apolipoprotein AI N terminus on the cell surface before lipidation and release of nascent high-density lipoprotein. Arterioscler. Thromb. Vasc. Biol..

[17] Czarnecka H., Yokoyama S. (1996). Regulation of cellular cholesterol efflux by lecithin:cholesterol acyltransferase reaction through nonspecific lipid exchange. J. Biol. Chem..

[18] Yuan Q., Zhao L. (2017). The Mulberry (*Morus alba* L.) Fruit—A Review of Characteristic Components and Health Benefits. J. Agric. Food Chem..

[19] Chen W., Li Y., Bao T., Gowd V. (2017). Mulberry Fruit Extract Affords Protection against Ethyl Carbamate-Induced Cytotoxicity and Oxidative Stress. Oxid. Med. Cell. Longev..

[20] Choi K.H., Lee H.A., Park M.H., Han J.S. (2016). Mulberry (*Morus alba* L.) Fruit Extract Containing Anthocyanins Improves Glycemic Control and Insulin Sensitivity via Activation of AMP-Activated Protein Kinase in Diabetic C57BL/Ksj-db/db Mice. J. Med. Food.

[21] Jung S., Lee M.S., Choi A.J., Kim C.T., Kim Y. (2019). Anti-Inflammatory Effects of High Hydrostatic Pressure Extract of Mulberry (Morus alba) Fruit on LPS-Stimulated RAW264.7 Cells. Molecules.

[22] Ju W.-T., Kwon O.-C., Lee M.-K., Kim H.-B., Sung G.-B., Kim Y.-S. (2017). Quali-quantitative analysis of flavonoids for mulberry leaf and fruit of ‘Suhyang’. Korean J. Environ. Agric..

[23] Yamamoto K. (2017). Food processing by high hydrostatic pressure. Biosci. Biotechnol. Biochem..

[24] Wang F., Du B.L., Cui Z.W., Xu L.P., Li C.Y. (2017). Effects of high hydrostatic pressure and thermal processing on bioactive compounds, antioxidant activity, and volatile profile of mulberry juice. Food Sci. Technol. Int..

[25] Friedewald W.T., Levy R.I., Fredrickson D.S. (1972). Estimation of the concentration of low-density lipoprotein cholesterol in plasma, without use of the preparative ultracentrifuge. Clin. Chem..

[26] Bligh E.G., Dyer W.J. (1959). A rapid method of total lipid extraction and purification. Can. J. Biochem. Physiol..

[27] Livak K.J., Schmittgen T.D. (2001). Analysis of Relative Gene Expression Data Using Real-Time Quantitative PCR and the 2(-Delta Delta C(T)) Method. Methods.

[28] Karathanasis S.K., Freeman L.A., Gordon S.M., Remaley A.T. (2017). The Changing Face of HDL and the Best Way to Measure It. Clin. Chem..

[29] Li Y., Bao T., Chen W. (2018). Comparison of the protective effect of black and white mulberry against ethyl carbamate-induced cytotoxicity and oxidative damage. Food Chem..

[30] Liu C.J., Lin J.Y. (2013). Anti-inflammatory effects of phenolic extracts from strawberry and mulberry fruits on cytokine secretion profiles using mouse primary splenocytes and peritoneal macrophages. Int. Immunopharmacol..

[31] Park Y.-S., Kang S.-S., Choi H.-J., Yang S.-J., Shon H.-H., Seo H.-H., Jeong J.-M. (2014). Effect of mulberry (Morus alba L.) extract on blood flow improvement. J. Korean Soc. Food Sci. Nutr..

[32] Jiang Y., Dai M., Nie W.-J., Yang X.-R., Zeng X.-C. (2017). Effects of the ethanol extract of black mulberry (Morus nigra L.) fruit on experimental atherosclerosis in rats. J. Ethnopharmacol..

[33] Liu L.K., Chou F.P., Chen Y.C., Chyau C.C., Ho H.H., Wang C.J. (2009). Effects of mulberry (Morus alba L.) extracts on lipid homeostasis in vitro and in vivo. J. Agric. Food Chem..

[34] Yan F., Dai G., Zheng X. (2016). Mulberry anthocyanin extract ameliorates insulin resistance by regulating PI3K/AKT pathway in HepG2 cells and db/db mice. J. Nutr. Biochem..

[35] Motamedi F., Nematbakhsh M., Monajemi R., Pezeshki Z., Talebi A., Zolfaghari B., Mansoori A., Saberi S., Dehghani A., Ashrafi F. (2014). Effect of pomegranate flower extract on cisplatin-induced nephrotoxicity in rats. J. Nephropathol..

[36] Widowati W., Ratnawati H., Mozefis T., Pujimulyani D., Yelliantty Y. (2013). Hypolipidemic and antioxidant effects of black tea extract and quercetin in atherosclerotic rats. Int. J. Med. Pharm. Sci. Eng..

[37] Loffredo L., Perri L., Nocella C., Violi F. (2017). Antioxidant and antiplatelet activity by polyphenol-rich nutrients: Focus on extra virgin olive oil and cocoa. Br. J. Clin. Pharmacol..

[38] Lonardo A., Sookoian S., Chonchol M., Loria P., Targher G. (2013). Cardiovascular and systemic risk in nonalcoholic fatty liver disease—Atherosclerosis as a major player in the natural course of NAFLD. Curr. Pharm. Des..

[39] Lu T.T., Makishima M., Repa J.J., Schoonjans K., Kerr T.A., Auwerx J., Mangelsdorf D.J. (2000). Molecular basis for feedback regulation of bile acid synthesis by nuclear receptors. Mol. Cell..

[40] Guan G., Dai P., Shechter I. (1998). Differential transcriptional regulation of the human squalene synthase gene by sterol regulatory element-binding proteins (SREBP) 1a and 2 and involvement of 5′ DNA sequence elements in the regulation. J. Biol. Chem..

[41] Yu L., Hammer R.E., Li-Hawkins J., Von Bergmann K., Lutjohann D., Cohen J.C., Hobbs H.H. (2002). Disruption of Abcg5 and Abcg8 in mice reveals their crucial role in biliary cholesterol secretion. Proc. Natl. Acad. Sci. USA.

[42] Pullinger C.R., Eng C., Salen G., Shefer S., Batta A.K., Erickson S.K., Verhagen A., Rivera C.R., Mulvihill S.J., Malloy M.J. (2002). Human cholesterol 7alpha-hydroxylase (CYP7A1) deficiency has a hypercholesterolemic phenotype. J. Clin. Investig..

[43] Li T., Matozel M., Boehme S., Kong B., Nilsson L.M., Guo G., Ellis E., Chiang J.Y. (2011). Overexpression of cholesterol 7alpha-hydroxylase promotes hepatic bile acid synthesis and secretion and maintains cholesterol homeostasis. Hepatology.

[44] Figge A., Lammert F., Paigen B., Henkel A., Matern S., Korstanje R., Shneider B.L., Chen F., Stoltenberg E., Spatz K. (2004). Hepatic overexpression of murine Abcb11 increases hepatobiliary lipid secretion and reduces hepatic steatosis. J. Biol. Chem..

[45] Paulusma C.C., Groen A., Kunne C., Ho-Mok K.S., Spijkerboer A.L., Rudi de Waart D., Hoek F.J., Vreeling H., Hoeben K.A., van Marle J. (2006). Atp8b1 deficiency in mice reduces resistance of the canalicular membrane to hydrophobic bile salts and impairs bile salt transport. Hepatology.

[46] Martinez-Fernandez P., Hierro L., Jara P., Alvarez L. (2009). Knockdown of ATP8B1 expression leads to specific downregulation of the bile acid sensor FXR in HepG2 cells: Effect of the FXR agonist GW4064. Am. J. Physiol. Gastrointest. Liver Physiol..

[47] Rohatgi A., Khera A., Berry J.D., Givens E.G., Ayers C.R., Wedin K.E., Neeland I.J., Yuhanna I.S., Rader D.R., de Lemos J.A. (2014). HDL cholesterol efflux capacity and incident cardiovascular events. N. Engl. J. Med..

[48] Saleheen D., Scott R., Javad S., Zhao W., Rodrigues A., Picataggi A., Lukmanova D., Mucksavage M.L., Luben R., Billheimer J. (2015). Association of HDL cholesterol efflux capacity with incident coronary heart disease events: A prospective case-control study. Lancet Diabetes Endocrinol..

[49] Hu Y.W., Zheng L., Wang Q. (2010). Regulation of cholesterol homeostasis by liver X receptors. Clinica Chimica Acta.

[50] Singaraja R.R., Fievet C., Castro G., James E.R., Hennuyer N., Clee S.M., Bissada N., Choy J.C., Fruchart J.C., McManus B.M. (2002). Increased ABCA1 activity protects against atherosclerosis. J. Clin. Investig..

[51] Brunham L.R., Singaraja R.R., Duong M., Timmins J.M., Fievet C., Bissada N., Kang M.H., Samra A., Fruchart J.C., McManus B. (2009). Tissue-specific roles of ABCA1 influence susceptibility to atherosclerosis. Arterioscler. Thromb. Vasc. Biol..

[52] Paszty C., Maeda N., Verstuyft J., Rubin E.M. (1994). Apolipoprotein AI transgene corrects apolipoprotein E deficiency-induced atherosclerosis in mice. J. Clin. Investig..

[53] Tietjen I., Hovingh G.K., Singaraja R., Radomski C., McEwen J., Chan E., Mattice M., Legendre A., Kastelein J.J., Hayden M.R. (2012). Increased risk of coronary artery disease in Caucasians with extremely low HDL cholesterol due to mutations in ABCA1, APOA1, and LCAT. Biochim. Biophys. Acta.

[54] Bartel D.P. (2004). MicroRNAs: Genomics, biogenesis, mechanism, and function. Cell.

[55] Marquart T.J., Allen R.M., Ory D.S., Baldan A. (2010). miR-33 links SREBP-2 induction to repression of sterol transporters. Proc. Natl. Acad. Sci. USA.

[56] Najafi-Shoushtari S.H., Kristo F., Li Y., Shioda T., Cohen D.E., Gerszten R.E., Naar A.M. (2010). MicroRNA-33 and the SREBP host genes cooperate to control cholesterol homeostasis. Science.

[57] Rayner K.J., Suarez Y., Davalos A., Parathath S., Fitzgerald M.L., Tamehiro N., Fisher E.A., Moore K.J., Fernandez-Hernando C. (2010). MiR-33 contributes to the regulation of cholesterol homeostasis. Science.

