# Supplementary material for: Mulberry Fruit Extract Promotes Serum HDL-Cholesterol Levels and Suppresses Hepatic microRNA-33 Expression in Rats Fed High Cholesterol/Cholic Acid Diet
Source: Nutrients. 2020 May 21;12(5):1499. doi: 10.3390/nu12051499 (PMC7284868; doi:10.3390/nu12051499)
Supplement: Supplementary file 1 [file nutrients-12-01499-s001.pdf]

nutrients

MDPI

Table S1. The compositions of experimental diets.

(g/ kg diet)

Component	NOR	HC	HM
Corn starch	150.0	150.0	150.0
Casein	200.0	200.0	200.0
Sucrose	500.0	485.0	481.0
Corn oil	50.0	50.0	50.0
Cellulose	50.0	50.0	50.0
Mineral mix [1)]	35.0	35.0	35.0
Vitamin mix [2)]	10.0	10.0	10.0
DL-Methionine	3.0	3.0	3.0
Choline bitartrate	2.0	2.0	2.0
Cholesterol	0.0	10.0	10.0
Cholic acid	0.0	5.0	5.0
Mulberry fruit extract	0.0	0.0	4.0
Total	1,000	1,000	1,000
Total calorie (kcal)	3774	3714	3698
Carbohydrates (% as kcal)	67.3	66.8	66.6
Protein (% as kcal)	19.0	19.3	19.4
Fat (% as kcal)	11.9	12.1	12.2

[1)] AIN-76 Mineral mix (mg/kg diet); [2)] AIN-76 Vitamin mix (mg/kg diet). NOR: normal diet, HC: high cholesterol/cholic acid diet containing 1% cholesterol and 0.5% cholic acid, HM; HC + 0.4% high hydrostatic pressure mulberry fruit. This experimental diet was formulated based on the AIN -76 diet composition.

Table S2. Primers used for quantitative real-time polymerase chain reaction (qRT-PCR).

Gene	GeneBank no.	Forward sequence (5′-3′)	Reverse sequence (5′-3′)	Product size (bp)
Abca1	NM_178095	CCCAATCCCAAACACTCCTT	TCTTCATCGTCCAGTTCCCA	107
Abcb11	NM_031760.1	CACTGGCCTTCTGGTATGGT	GCTTGTAGCCGTCTCCTGAC	225
Abcg5	NM_053754	ATGAGTGAGCTGCCCTTTCT	CGCTGAAGGACACATTCAGG	142
Abcg8	NM_130414	GAGAACTTTGTCCCGCCTGG	AGATAGGGGTGCCAGACGTC	122
ApoA-1	NM_012738	CTGGGTTCAACTGTTGGTCG	GGGCTGCATCTTCTGTTTCA	147
Atp8b1	NM_001106140.1	TACGGTCATGGTGGACAGAA	ATGCTACCTTCTGGGGTCCT	237
β-actin	NM_031144	GGCACCACACTTTCTACAAT	AGGTCTCAAACATGATCTGG	123
Cyp7a1	NM_012942	TGTTCTGTGTTCACTTTCTG	ACTCGGTAACAGAAGGCATA	126
Fxr	NM_021745.1	CGCCGTGTACAAGTGTAAGA	TTCACTGTCTGATCCGCATG	109
Lcat	NM_017024	TAACAATGGGTATGTGCGGG	GCCAAGGCTGTGTCCAATAA	163
Lxr-α	NM_031627	GACTTCGAGTCACGCCTTGG	GTCCTCCCTGCTCAGCTGTA	161
Shp	NM_057133.1	CCTTGGATGTCCTAGGCAAG	CACCACTGTTGGGTTCCTCT	213
Sreb2	NM_001033694.1	GACAGGATGAAGCCACCATT	GGCAAGAGACCTGAGTCCTG	214

Abca1, ATP-binding cassette, subfamily A member 1; Abcb11, ATP binding cassette subfamily B member 11; Abcg5, ATP-binding cassette subfamily G member 5; Abcg8, ATP-binding cassette subfamily G member 8; ApoA-1, apolipoprotein A1; Atp8b1, ATPase phospholipid transporting 8B1; Cyp7a1, cholesterol 7 alpha-hydroxylase; Fxr, farnesoid x receptor; Lcat, lecithin cholesterol acyltransferase; Lxr-α, liver X receptor α; Shp, small heterodimer partner; Sreb2, sterol regulatory element binding transcription factor 2

© 2020 by the authors. Submitted for possible open access publication under the terms and conditions of the Creative Commons Attribution (CC BY) license (http://creativecommons.org/licenses/by/4.0/).
